# Antimicrobial susceptibility testing of *Neisseria gonorrhoeae* isolates in Pakistan by Etest compared to Calibrated Dichotomous Sensitivity and Clinical Laboratory Standards Institute disc diffusion techniques

**DOI:** 10.1186/s12866-016-0707-6

**Published:** 2016-10-10

**Authors:** Pushpa Bhawan Mal, Kauser Jabeen, Joveria Farooqi, Magnus Unemo, Erum Khan

**Affiliations:** 1 Section of Pathology and Laboratory Medicine, Clinical Microbiology Aga Khan University, Stadium Road, P.O. Box 3500, Karachi, 74800 Pakistan; 2 WHO Collaborating Centre for Gonorrhoea and other STIs, Department of Laboratory Medicine, Clinical Microbiology, Faculty of Medicine and Health, Örebro University, Örebro, Sweden

**Keywords:** Antimicrobial surveillance, *Neisseria gonorrhoeae*, CDS, CLSI, Disc diffusion, Etest

## Abstract

**Background:**

Accurate detection of *Neisseria gonorrhoeae* antimicrobial resistance is essential for appropriate management and prevention of spread of infection in the community. In this study Calibrated Dichotomous Sensitivity (CDS) and Clinical Laboratory Standards Institute (CLSI) disc diffusion methods were compared with minimum inhibitory concentration (MIC) by Etest in *Neisseria gonorrhoeae* isolates from Karachi, Pakistan. CDS and CLSI disc diffusion techniques, and Etest for ceftriaxone, penicillin G, spectinomycin and ciprofloxacin against 100 isolates from years 2012–2014 were performed. Due to lack of CLSI breakpoints for azithromycin, it was interpreted using cut-offs from British Society of Antimicrobial Chemotherapy (BSAC). Due to lack of low concentration tetracycline discs, tetracycline was tested with CLSI disc diffusion and Etest only. Comparisons were based on the identified susceptibility, intermediate susceptibility and resistance (SIR) categories using the different methods. Complete percent agreement was percentage agreement achieved when test and reference method had identical SIR-category. Essential percent agreement was percentage agreement when minor discrepancies were disregarded.

**Results:**

There was 100 % and 99 % overall essential agreement and 50 % versus 23 % overall complete agreement by CDS and CLSI methods, respectively, with MICs for all tested antibiotics. Using either method, there was 100 % complete agreement for ceftriaxone and spectinomycin. There was 90 % versus 86 % complete agreement for ciprofloxacin, and 60 % and 75 % for penicillin using CDS and CLSI method, respectively. Essential agreement of 99 % and complete agreement of 62 % was found for tetracycline with CLSI method. There was 100 % essential and complete agreement by CDS, BSAC and Etest for azithromycin.

**Conclusion:**

No major errors with regard to identified SIR-categories were found for penicillin, ciprofloxacin, ceftriaxone and spectinomycin using CLSI and CDS methods. All isolates were susceptible to ceftriaxone and spectinomycin, and 99 % to azithromycin. In low-resource settings, both the CLSI and CDS disc diffusion techniques might be used for susceptibility testing of gonococcal isolates. However, these methods require considerable standardization and quality controls for adequate levels of reproducibility and correct interpretation to reflect appropriately the MIC values of the different antimicrobials. New, emerging, or rare resistance should be confirmed by MIC determination.

## Background

Treatment of gonorrhea is compromised due to global emergence and dissemination of *Neisseria gonorrhoeae* strains that are resistant to most antimicrobial agents available for treatment [1]. Accurate detection of antimicrobial resistance is essential for appropriate management of gonorrhea and prevention of spread of infection as well as complications in individual patients [2].

There are several methods available for antimicrobial susceptibility testing in *N. gonorrhoeae.* These include disc diffusion methods and/or breakpoints recommended by the Clinical and Laboratory Standards Institute (CLSI) [3], British Society for Antimicrobial Chemotherapy (BSAC) [4] and European Committee on Antimicrobial Susceptibility Testing (EUCAST) [5], and agar dilution and the Etest techniques that determine the minimum inhibitory concentration (MIC) of antimicrobials [3]. Many laboratories in less-resourced settings use high-content disc diffusion method recommended by the CLSI due to its ease and low cost. However, disc diffusion method might not accurately detect all gonococcal strains with decreased susceptibility or low-level resistance to extended-spectrum cephalosporins (ESCs) [6]. Calibrated Dichotomous Sensitivity method (CDS) using low content disc diffusion methodology has, in situations when MIC determination cannot be performed, been advocated by the World Health Organization (WHO) programs for *N. gonorrhoeae* antimicrobial resistance surveillance [7]. Use of a 30 μg ceftriaxone disc as recommended by the CLSI has been reported to be ineffective to detect decreased susceptibility and low-level resistance to ESCs in *N. gonorrhoeae* strains [6]. Therefore use of a ceftriaxone 0.5 μg disc has been advocated by the CDS-based WHO based programs for *N. gonorrhoeae* antimicrobial resistance surveillance. The lower potency ceftriaxone disc can more effectively detect increases at the lower MIC values [7]. In the CDS technique, it was also recently recommended to additionally use a 10 μg cefpodoxime disc for detection of decreased susceptibility and resistance to ESCs. The 10 μg cefpodoxime disc was shown to be an effective screening method for detection of gonococcal strains containing a *penA* mosaic allele encoding a mosaic penicillin-binding protein 2 (PBP2), which can result in decreased susceptibility or resistance to ESCs [8]. *N. gonorrhoeae* strains with decreased susceptibility or resistance to ESCs have not yet been reported from Pakistan [9]. However, mainly the CLSI recommended disc diffusion method has been used and, accordingly, it is essential to appropriately evaluate this method on the *N. gonorrhoeae* strains circulating in Pakistan.

The objective of the present study was to evaluate the performance of CDS and CLSI disc diffusion techniques in assessing susceptibilities of *N. gonorrhoeae* to ceftriaxone, penicillin G, spectinomycin, and ciprofloxacin. MIC determination using the Etest was applied as a reference method. Due to the non-availability of low potency tetracycline discs, tetracycline was compared using CLSI and reference method only. Furthermore, azithromycin was tested using BSAC and CDS methods only as no azithromycin interpretative breakpoints are stated by the CLSI. Only two studies have previously performed a head to head comparison of the two main disc diffusion methods (CDS and CLSI) with the Etest [10, 11].

## Methods

This prospective descriptive cross-sectional study was conducted at the Department of Pathology and Laboratory Medicine, Aga Khan University Hospital (AKUH), Karachi, Pakistan. The laboratory is a sentinel site for surveillance of gonococcal resistance and regularly participates in international gonococcal external quality assessment program. One hundred consecutive *N. gonorrhoeae* isolates cultured from urethral swabs (*n* = 85), high vaginal and cervical swabs (*n* = 14) and conjunctiva swab (*n* = 1) from January 2012 to February 2014 were included in the study. The study was approved by the Ethical Review Committee of university (Exemption #2365-Pat-12). *N. gonorrhoeae* isolates were identified by conventional tests using standard protocol including colony morphology, Gram staining, oxidase test, sugar utilization and Remel RapidNH® Panel (BioMérieux, France).

### Antimicrobial susceptibility testing

Antimicrobial susceptibility testing was performed and interpreted according to the CDS [7], CLSI [3], and only for azithromycin BSAC [4] criteria using disc diffusion methods, in strict accordance to the instructions from the manufacturer. Susceptibility with the CLSI method was performed as routine clinical laboratory work. Isolates were subsequently stored at −80 °C in glycerol buffered phosphate. The isolates were later revived and the CDS and Etest methods were performed simultaneously in four batches. MIC determination was performed using Etest strips as specified by the manufacturer (AB Biodisk, Stockholm, Sweden) against ceftriaxone, penicillin G, spectinomycin, ciprofloxacin, azithromycin and tetracycline. Finally, *N. gonorrhoeae* isolates were also tested for β-lactamase production by the chromogenic cephalosporin method using nitrocefin freeze-dried powder (Oxoid, Hampshire, UK).

### Inoculum preparation

A homogenous suspension of 0.5 McFarland turbidity was made from fresh culture (24 h) on GC agar base (GC agar base medium, Becton Dickinson, UK) with 1 % BBL IsoVitalex Enrichment (Becton Dickinson, France). For all four methods (CLSI, CDS, BSAC and Etest), inoculum was prepared by emulsifying single large or 2–3 small colonies in 2.5 mL sterile saline (0.85 %). This suspension was used within 15 min. All plates for antimicrobial susceptibility testing were incubated at 35–37 °C in 5 % CO_2_-enriched humid atmosphere for 20-h [3, 7].

### CDS disc diffusion method

Due to non-availability of chocolate Columbia agar, antibiotic susceptibility testing was performed on GC agar base (GC agar base medium, Becton Dickinson, UK) with 1 % BBL IsoVitalex Enrichment (Becton Dickinson, France) with the following low concentration antibiotic discs (Oxoid): ceftriaxone (0.5 μg), cefpodoxime (10 μg), penicillin G (0.5 IU), spectinomycin (100 μg), ciprofloxacin (1 μg), azithromycin (15 μg) and nalidixic acid (30 μg). The use of this alternate agar medium for susceptibility testing did not affect the results of the quality control strains and, accordingly, the results were considered valid. The results were interpreted by measuring the annular radius of the inhibition zones and categorized as susceptible (S), intermediate susceptible (I) and resistant (R) [7]. Cefpodoxime disc (10 μg) was used as screening method for detection of decreased susceptibility or resistance to ESC [8]. Nalidixic acid was used as a marker for detection of isolates with decreased susceptibility to ciprofloxacin. Due to non-availability of low potency tetracycline discs, CDS disc diffusion technique was not performed for tetracycline.

### CLSI disc diffusion method

Antibiotic susceptibility testing was performed on GC agar base (GC agar base medium, Becton Dickinson, UK) with 1 % BBL IsoVitalex Enrichment (Becton Dickinson, France) with the following high concentration discs (Oxoid): ceftriaxone (30 μg), penicillin (10 IU), spectinomycin (100 μg), ciprofloxacin (5 μg), nalidixic acid (30 μg) and tetracycline (30 μg). The results were interpreted by measuring the inhibition zone diameters and categorized as susceptible, intermediate susceptible and resistant [3].

### BSAC disc diffusion method

Antibiotic susceptibility testing for azithromycin (15 μg) was performed on GC agar base (GC agar base medium, Becton Dickinson, UK) with 1 % BBL IsoVitalex Enrichment (Becton Dickinson, France). The results were interpreted by measuring inhibition zone diameters and categorized as susceptible, intermediate susceptible or resistant [4].

### Etest method

The Etest method was used as reference method. MICs of ceftriaxone, penicillin G, spectinomycin, ciprofloxacin, azithromycin and tetracycline were determined by the Etest method (AB Biodisk), according to manufacturer’s instructions, on GC agar base (GC agar base medium, Becton Dickinson, UK) with 1 % BBL IsoVitalex Enrichment (Becton Dickinson, France). The results were interpreted in SIR categories according to the CLSI criteria [3].

### Quality control strains

*N. gonorrhoeae* reference strain ATCC 49226 and the 2008 WHO reference strains F, G, K, L, M, N, O, and P [12] were used as quality controls for the disc diffusion methods. MICs using the Etest for these strains were tested against ceftriaxone only (Table 1).

**Table 1:** Mean annular radius and expected ranges of WHO *Neisseria gonorrhoeae* reference strains (F, G, K, L, M, N, O and P)

Methods	Antibiotics tested		F	G	K	L	M	N	O	P
CDS	Penicillin G	Mean annular radius (range)	13.2 (12–15)	5.5 (3–7)	00	00	00	00	00	6 (5–7)
		Expected result	>9 (S)	3–9 (I)	<3 (R)	<3 (R)	<3 (R)	<3 (R)	<3 (R)	3–9 (I)
		β-lactamase production	No	No	No	No	Yes	Yes	Yes	No
		Expected result	No	No	No	No	Yes	Yes	Yes	No
	Ciprofloxacin	Mean annular radius (range)	15 (14–17)	7.7 (7–9)	00	00	3.2 (02–4)	00	13.5 (12–15)	14.2 (13–16)
		Expected result	≥11 (S)	6–10 (I)	<6 (R)	<6 (R)	<6 (R)	<6 (R)	≥11 (S)	≥11 (S)
	Spectinomycin	Mean annular radius (range)	11 (10–11)	10.2 (9–11)	16 (15–18)	9.5 (8–11)	11 (9–13)	13.5 (10–17)	00	13 (11–15)
		Expected result	≥6 (S)	≥6 (S)	≥6 (S)	≥6 (S)	≥6 (S)	≥6 (S)	<6 (R)	≥6 (S)
	Azithromycin	Mean annular radius (range)	12.5 (10–15)	14 (12–16)	13.5 (12–15)	11 (9–13)	12 (9–15)	11.5 (10–13)	13 (12–15)	1.5 (0–4)
		Expected result	≥8 (S)	≥8 (S)	≥8 (S)	≥8 (S)	≥8 (S)	≥8 (S)	≥8 (S)	<8 (R)
	Ceftriaxone	Mean annular radius (range)	14.2 (12–16)	14.7 (14–16)	6.7 (6–8)	5.5 (5–7)	13 (12–15)	13.2 (12–15)	13.2 (13–15)	13.7 (13–15)
		Expected result	≥10 (S)	≥10 (S)	5–9 (DS)	5–9 (DS)	≥10 (S)	≥10 (S)	≥10 (S)	≥10 (S)
MIC by Etest	Ceftriaxone^a^	Etest MIC ranges	<0.002	0.004–0.008	0.064–0.125	0.125–0.25	0.008–0.016	0.002–0.004	0.016–0.032	0.004–0.008
		Expected result	<0.002 (S)	0.004–0.016 (S)	0.032–0.125 (DS)	0.064–0.25 (DS)	0.008–0.032 (S)	0.002–0.008 (S)	0.016–0.064 (S)	0.002–0.008 (S)

^a^MICs for control strains was performed only for ceftriaxone. *DS* Decreased susceptibility, *S* Sensitive, *I* intermediate, *R* resistant

## Statistical analysis

Data were analyzed using Stata 12 and Microsoft Excel 2010. The SIR-categories using the CDS and CLSI disk diffusion methods were compared with the SIR-categories obtained by the reference method, i.e., the Etest. Rates of discrepancies for each antibiotic were determined into three categories minor [test result showed R or S and reference results showed I or test results showed I and reference results showed R or S], major (S interpreted as R) and very major (R interpreted as S) [13].

Complete and essential percent agreement between the reference and test method was calculated. The complete percent agreement value was the proportion of isolates with identical SIR-category by both test and reference methods. The essential percent agreement value was proportion of isolates with similar results by both reference and test method when minor errors (as defined above) were disregarded [13]. Kappa value was calculated as the statistical measure of SIR-agreement between reference (Etest) and evaluated (CLSI or CDS) methods. Kappa value below zero depicted poor, 0.00–0.20 showed slight agreement, 0.21–0.40 fair, 0.41–0.60 moderate, 0.61–0.80 substantial, and 0.81–1.00 very high agreement.

## Results

Antimicrobial susceptibility testing of 100 *N. gonorrhoeae* isolates was performed by two disc diffusion techniques and Etest (reference method) for ceftriaxone, penicillin G, spectinomycin, ciprofloxacin and tetracycline. The inhibition zone diameters and MICs of the WHO reference strains (F, G, K, L, M, N, O and P) were within the acceptable range for these quality control strains (Table 1). All (100 %) isolates were susceptible to ceftriaxone and spectinomycin by all three methods. Using Etest, 99 %, 12 %, 8 % and 0 % isolates were susceptible to azithromycin, tetracycline, penicillin G and ciprofloxacin, respectively (Table 2). Table 3 shows comparison of discrepancies and agreement of CDS and CLSI disc diffusion method with Etest as well as kappa scores for five different antibiotics. The rates of discrepancies for each antibiotic were different for both the CDS and CLSI technique on comparison with Etest method as shown in Table 3. An overall essential agreement of 99 % and an overall complete agreement of 23 % were found between CLSI disc diffusion method and Etest. An overall essential agreement of 100 % and an overall complete agreement of 50 % were found between CDS disc diffusion method and Etest. Statistically kappa scores for penicillin G showed that CDS method had fair while CLSI method had moderate level of agreement with Etest. For ciprofloxacin, CDS method had moderate agreement while CLSI method had slight agreement with Etest. For tetracycline, CLSI method had fair level of agreement (Table 3). For ceftriaxone and spectinomycin; kappa value could not be calculated because there was only one category i.e. susceptible. Accordingly, the complete percent agreement of the CDS and CLSI method with Etest for ceftriaxone and spectinomycin was 100 %.

**Table 2:** Antimicrobial susceptibility results of *Neisseria gonorrhoeae* isolates by CDS/CLSI disc diffusion and MIC by Etest (*n* = 100)

Methods	Penicillin G %			Ciprofloxacin %			Ceftriaxone %			Tetracycline %			Spectinomycin %			Azithromycin %		
	S	I	R	S	I	R	S	I	R	S	I	R	S	I	R	S	I	R
Etest MIC	08	49	43	00	14	86	100	00	00	12	37	51	100	00	00	99	00	01
CLSI/BSAC^a^ MIC break points (μg/ml)	≤0.06	0.12-1	≥2	≤0.06	0.12-0.5	≥1	≤0.25	-	-	≤0.25	0.5–1	≥2	≤32	64	≥128	≤0.25	0.5	≥0.5
CDS disc diffusion	10	13	77	00	20	80	100	00	00	NP	NP	NP	100	00	00	99	00	01
CDS annular radius break points (mm)	>9	3-9	<3	≥11	6-10	<6	≥10	-	5-9	NP	NP	NP	≥6	-	<6	≥8	-	<8
CLSI disc diffusion	04	34	62	00	04	96	100	00	00	03	40	57	100	00	00	99	00	01
CLSI/BSAC^a^ zone diameter break points (mm)	≥47	27-46	≤26	>41	28-40	≤27	≥35	-	-	≥38	31-37	<30	≥18	15-17	≤4	≥28	-	≤27

*S* sensitive, *I* intermediate, *R* resistant

*NP* not performed

^a^BSAC breakpoints were used for Azithromycin

**Table 3:** Comparison of discrepancies and agreement between the CDS, CLSI and Etest method for 100 *Neisseria gonorrhoeae* isolates

Antibiotics	No. of discrepancies						% Agreement				Kappa value	
	Minor		Major		Very major		Complete		Essential			
Methods	CDS	CLSI	CDS	CLSI	CDS	CLSI	CDS	CLSI	CDS	CLSI	CLSI	CDS
Ceftriaxone	00	00	00	00	00	00	100	100	100	100	-	-
Spectinomycin	00	00	00	00	00	00	100	100	100	100	-	-
Penicillin G	40	25	00	00	00	00	60	75	100	100	0.5652	0.3435
Ciprofloxacin	10	14	00	00	00	00	90	86	100	100	0.0990	0.6718
Tetracycline	-^a^	37	-^a^	01	-^a^	00	-^a^	62	-^a^	99	0.2963	-^a^
Overall	50	76	00	01	00	00	50	23	100	99	-	-

^a^Not tested

Minor error: test result showed resistant or susceptible and reference results showed intermediate or test results showed intermediate and reference results showed resistant or susceptible

Major error: test result showed susceptible and reference results showed resistant

Very major error: test result showed resistant and reference results susceptible

Complete percent agreement value was percentage agreement achieved when the test and reference method had identical SIR-category of result

Essential percent agreement value was percentage agreement obtained between the reference and test method when minor discrepancies were disregarded

Table 4 shows SIR agreement rates for penicillin, azithromycin and ciprofloxacin using CDS method and penicillin, tetracycline, azithromycin and ciprofloxacin using CLSI method against reference method. This table also describes the number of concordant and discordant results using these two methods. Majority of discordant results were seen with penicillin using both methods and with tetracycline using CLSI method. Ciprofloxacin discordance rates were comparable by both CDS and CLSI methods. The details of each antibiotic results are discussed separately below.

**Table 4:** SIR agreement rate of *Neisseria gonorrhoeae* isolates by CDS/CLSI disc diffusion against MIC by Etest (All 100 isolates were sensitive to ceftriaxone and spectinomycin by all three methods therefore are not shown in this table)

	Category	Etest			Complete agreement	
					Concordant results	Discordant Results
		S	I	R		
CDS						
Penicillin G	S	06	04	00	60	40
	I	02	11	00		
	R	00	34	43		
Ciprofloxacin	S	00	00	00	90	10
	I	00	12	08		
	R	00	02	78		
Azithromycin	S	99	00	00	100	00
	I	00	00	00		
	R	00	00	01		
CLSI						
Penicillin G	S	04	00	00	75	25
	I	04	29	01		
	R	00	20	42		
Ciprofloxacin	S	00	00	00	86	14
	I	00	02	02		
	R	00	12	84		
Tetracycline	S	01	02	00	62	37
	I	10	20	10		
	R	01	15	41		
Azithromycin^a^	S	99	00	00	100	00
	I	00	00	00		
	R	00	00	01		

SIR: Sensitive, intermediate, resistant

Tetracycline was tested with CLSI method only

^a^BSAC break points were used for Azithromycin

### Penicillin G

By the Etest, 08 %, 49 % and 43 % of isolates were interpreted as susceptible, intermediate susceptible and resistant, respectively (Table 2). Of 43 penicillin G resistant isolates, 41 were β-lactamase positive and two had chromosomally mediated resistance. On comparison of Etest with CDS and CLSI disc diffusion methods, minor discrepancies were observed at 40 % and 25 %, respectively (Table 3; details of discordance are shown in Table 4). Major and very major discrepancies were not found with any of the disc diffusion methods. The complete percent agreement of the CDS and CLSI method with Etest for penicillin G was 60 % and 75 % respectively, and the essential agreement was 100 %.

### Ciprofloxacin

None of the isolates were susceptible to ciprofloxacin by Etest. The number of intermediate susceptible and resistant isolates were 14 (14 %) and 86 (86 %), respectively (Table 3). Out of 86 resistant isolates, 48 (55.8 %) had a high-level resistance (defined as MIC ≥4 μg/ml).

On comparison of Etest with CDS and CLSI disc diffusion methods, minor discrepancies were observed at 10 % and 14 %, respectively (Tables 3, 4). Complete agreement for the CDS and CLSI method was 90 % and 86 %, respectively. No major and very major discrepancies were observed.

### Tetracycline

By Etest 12 %, 37 % and 51 % of isolates were interpreted as susceptible, intermediate susceptible and resistant, respectively (Table 2). Out of 51 resistant isolates, 28(54.9 %) were tetracycline resistant *N. gonorrhoeae* (TRNG) (MIC ≥16 μg/ml). On comparison of the CLSI disc diffusion technique with the Etest, 1 % major and 37 % minor discrepancies were found (Table 3). The complete and essential percentage agreement for the CLSI technique was 62 % and 99 %, respectively.

### Azithromycin

Ninety-nine percent and 01 % of isolates were interpreted as susceptible and resistant, respectively, by the Etest, BSAC and CDS disc diffusion methods (Table 2). 100 % complete agreement and essential agreement were found for both the BSAC and CDS method when compared with the Etest.

## Discussion

Present study reports a head to head comparison of the CLSI and CDS disc diffusion methods with the Etest (MIC-based reference method) for antimicrobial susceptibility testing of *N. gonorrhoeae*. A poor overall agreement (23 %) between the CLSI disc diffusion method and the Etest was seen. Discrepancies were most frequent for tetracycline, where 37 minor errors and 1 major error were noted with a complete agreement of 62 % with the Etest. These results are concordant with findings of Singh et al. from India, where an overall agreement of 49.5 % was reported between the CLSI method and reference method [10]. In this previous study [10] also, one of the main reason of the poor performance of the CLSI method was the low agreement for tetracycline (75 %) with the reference method. When excluding tetracycline from the analysis, the overall agreement of the CLSI method with the reference method increased from 23 % to 61 % in our study and to 49.5 % to 75 % in the study by Singh et al. [10]. In contrast, Khaki et al. reported excellent agreement of the CLSI method with the reference method [11].

In the current study, an overall agreement of 50 % was found between the CDS method and the reference method. Singh et al. reported an overall agreement of 82 % between CDS method and the reference method [10]. Khaki et al. also reported excellent agreement of CDS with the reference method [11]. We could not compare our results with these studies because we did not perform CDS testing of tetracycline due to non-availability of low concentration tetracycline discs.

Our findings for ceftriaxone and spectinomycin were consistent with those of Singh et al. who reported 100 % complete agreement of spectinomycin by both methods and 98.6 % complete agreement of ceftriaxone by CLSI and 98.3 % by CDS technique [10]. Khaki et al. reported 100 % complete agreement of both the CDS and CLSI methods and the reference method for spectinomycin and ceftriaxone [11]. For penicillin G higher proportion of isolates were labeled as resistant by the CDS method in comparison to reference method. These results were similar to Khaki et al. who also reported that the CDS method interpreted intermediate isolates as resistant to penicillin [11]. Bala et al. reported moderate level of agreement between the CDS method and reference method for penicillin, that is, 28 % minor errors by the CDS method [14].

For ciprofloxacin our results are consistent with Singh et al. and Bala et al. Accordingly, Singh et al. reported a complete agreement of 88.5 % for the CLSI method and 92.9 % for the CDS method with reference method and Bala et al. reported a complete agreement of 79.8 % for the CDS method with reference method [10, 14]. In contrast, Khaki et al. reported 100 % agreement by both methods [11].

No discrepancy was observed for azithromycin between the CDS, BSAC and reference methods. Due to the lack of azithromycin SIR break-points stated by the CLSI, the CLSI method was not evaluated for this antimicrobial in the current study or previous studies by Singh et al. and Bala et al. [10, 14].

The current study observed an overall essential agreement of 100 % and 99 % with CDS and CLSI technique, respectively, with the reference method. Only one isolate showed a major error in the tetracycline reporting by the CLSI method in current study. In contrast, Singh et al. reported 16 major errors by CLSI method in tetracycline reporting and 2 major errors by CDS method [10].

In less-resourced settings, where the preferred MIC-testing cannot be performed, Singh et al. reported that the CDS disc diffusion method was preferred to the CLSI disc diffusion method for antimicrobial susceptibility testing of *N. gonorrhoeae* [10]. However, Khaki et al. recommended the CLSI method because it was more accurate and more feasible compared to the CDS method. In the current study, no major errors were identified for penicillin G, ciprofloxacin, ceftriaxone and spectinomycin testing by any of the methods.

### Limitations

Low concentration disc for tetracycline was not tested so its comparison with CDS method was not possible. Recommended media like Columbia agar for CDS and 5 % defibrinated horse blood agar for BSAC methods were not used due to non-availability of these media. However the techniques were optimized on the media used in current study by using WHO quality control strains for CDS technique and ATCC 49226 for BSAC technique. Since 100 % of investigated isolates were susceptible to ceftriaxone and spectinomycin, and 99 % to azithromycin, agreement across all categories could not be assessed.

## Conclusion

In less-resourced settings, both the CLSI and CDS disc diffusion techniques might be used for susceptibility testing of gonococcal isolates. However results of disc diffusion methods for tetracycline and for isolates approaching SIR breakpoints for ceftriaxone, spectinomycin and azithromycin should be interpreted with caution. Furthermore, the disc diffusion methods require considerable standardization and appropriate quality control measures to attain an adequate level of reproducibility and correct interpretation, reflecting the MIC values of the different antimicrobials. New, emerging, or rare resistance should always be confirmed by MIC determination. Finally, due to the non-availability of low antimicrobial concentration discs in the country a wide implementation of CDS disc diffusion method in Pakistan could be challenging.

## Abbreviations

ESCs: extended spectrum cephalosporins
CDS: calibrated dichotomous sensitivity
WHO: World Health Organization
CLSI: Clinical Laboratory Standards Institute
BSAC: British Society of Antimicrobial Chemotherapy
MIC: minimum inhibitory concentration
SIR: susceptibility, intermediate susceptibility and resistance categories

## References

[1] Unemo M, Shafer WM (2014). Antimicrobial resistance in *Neisseria gonorrhoeae* in the 21st century: past, evolution, and future. Clin Microbiol Rev.

[2] World Health Organization: Global action plan to control the spread and impact of antimicrobial resistance in *Neisseria gonorrhoeae*. Geneva 2012:1–36.

[3] Clinical and Laboratory Standards Institute:Performance standards for antimicrobial suspectibility testing; twenty second information supplement. CLSI document M100-S22. vol. 32; 2012.

[4] Andrews J (2009). BSAC standardized disc susceptibility testing method (version 8). J Antimicrob Chemother.

[5] Testing ECoAS: Breakpoint tables for interpretation of MICs and zone diameters. Version 3.1, 2013. Online at http://www.eucast.org 2013.

[6] World Health Organization: Consultation on the Strategic Response to the Threat of Untreatable *Neisseria gonorrhoeae* and Emergence of Cephalosporin Resistance in *Neisseria gonorrhoeae*. Manila, WHO; 2010. In: WHO Meeting Report: 2010; 2010.

[7] Bell SM: The CDS disc method of antibiotic sensitivity testing (calibrated dichotomous sensitivity test). Pathology 1975, 7(4 Suppl):Suppl 1–48. 10.3109/00313027509082602 772573

[8] Limnios A, Tapsall J, Kahlmeter J, Hogan T, Ray S, Lam A, Unemo M (2011). Cefpodoxime 10 mug disc screening test for detection of *Neisseria gonorrhoeae* with mosaic PBP2 and decreased susceptibility to extended-spectrum cephalosporins for public health purposes. APMIS.

[9] Zafar A, Jabeen K (2007). Antimicrobial resistance in *Neisseria gonorrhoeae* and limited treatment options. J Pak Med Assoc.

[10] Singh V, Bala M, Kakran M, Ramesh V. Comparative assessment of CDS, CLSI disc diffusion and Etest techniques for antimicrobial susceptibility testing of *Neisseria gonorrhoeae*: a 6-year study. BMJ Open. 2012;2(4):1-7. 10.1136/bmjopen-2012-000969 PMC3391364 22761285

[11] Khaki P, Sharma A, Bhalla P (2014). Comparison of two disc diffusion methods with minimum inhibitory concentration for antimicrobial susceptibility testing of *Neisseria gonorrhoeae* isolates. Ann Med Health Sci Res.

[12] Unemo M, Fasth O, Fredlund H, Limnios A, Tapsall J (2009). Phenotypic and genetic characterization of the 2008 WHO *Neisseria gonorrhoeae* reference strain panel intended for global quality assurance and quality control of gonococcal antimicrobial resistance surveillance for public health purposes. J Antimicrob Chemother.

[13] Food, Administration D: Class II special controls guidance document: antimicrobial susceptibility test (AST) systems; guidance for industry and FDA. Food and Drug Administration, Rockville, MD. 2007. http://www.fda.gov/cdrh/oivd/guidance/631 html.

[14] Bala M, Ray K, Gupta SM (2005). Comparison of disc diffusion results with minimum inhibitory concentration (MIC) values for antimicrobial susceptibility testing of *Neisseriagonorrhoeae*. Indian J Med Res.

